# Whole-genome-based characterization of *Campylobacter jejuni* from human patients with gastroenteritis collected over an 18 year period reveals increasing prevalence of antimicrobial resistance

**DOI:** 10.1099/mgen.0.000941

**Published:** 2023-02-21

**Authors:** Giovanni Ghielmetti, Helena M. B. Seth-Smith, Tim Roloff, Nicole Cernela, Michael Biggel, Roger Stephan, Adrian Egli

**Affiliations:** ^1^​ Institute for Food Safety and Hygiene, Section of Veterinary Bacteriology, University of Zurich, Zurich, Switzerland; ^2^​ Applied Microbiology Research, Department of Biomedicine, University of Basel, Basel, Switzerland; ^3^​ Clinical Bacteriology and Mycology, University Hospital Basel, Basel, Switzerland; ^4^​ Swiss Institute for Bioinformatics, Basel, Switzerland; ^5^​ Institute for Food Safety and Hygiene, University of Zurich, Zurich, Switzerland

**Keywords:** antimicrobial resistance, *Campylobacter jejuni*, chromosomal cassette, Switzerland, time-scaled phylogenetic analysis, whole-genome sequencing

## Abstract

Campylobacteriosis is the most common cause of acute gastrointestinal bacterial infection in Europe, with most infections linked to the consumption of contaminated food. While previous studies found an increasing rate of antimicrobial resistance (AMR) in *Campylobacter* spp. over the past decades, the investigation of additional clinical isolates is likely to provide novel insights into the population structure and mechanisms of virulence and drug resistance of this important human pathogen. Therefore, we combined whole-genome sequencing and antimicrobial-susceptibility testing of 340 randomly selected *Campylobacter jejuni* isolates from humans with gastroenteritis, collected in Switzerland over an 18 year period. In our collection, the most common multilocus sequence types (STs) were ST-257 (*n*=44), ST-21 (*n*=36) and ST-50 (*n*=35); the most common clonal complexes (CCs) were CC-21 (*n*=102), CC-257 (*n*=49) and CC-48 (*n*=33). High heterogeneity was observed among STs, with the most abundant STs recurring over the entire study period, while others were observed only sporadically. Source attribution based on ST assigned more than half of the strains to the ‘generalist’ category (*n*=188), 25  % as ‘poultry specialist’ (*n*=83), and only a few to ‘ruminant specialist’ (*n*=11) or ‘wild bird’ origin (*n*=9). The isolates displayed an increased frequency of AMR from 2003 to 2020, with the highest rates of resistance observed for ciprofloxacin and nalidixic acid (49.8 %), followed by tetracycline (36.9 %). Quinolone-resistant isolates carried chromosomal *gyrA* mutations T86I (99.4 %) and T86A (0.6 %), whereas tetracycline-resistant isolates carried *tet(O*) (79.8 %) or mosaic *tetO/32/O* (20.2 %) genes. A novel chromosomal cassette carrying several resistance genes, including *aph(3')-III*, *satA* and *aad*(6), and flanked by insertion sequence elements was detected in one isolate. Collectively, our data revealed an increasing prevalence of resistance to quinolones and tetracycline in *C. jejuni* isolates from Swiss patients over time, linked to clonal expansion of *gyrA* mutants and acquisition of the *tet(O*) gene. Investigation of source attribution suggests that infections are most likely related to isolates from poultry or generalist backgrounds. These findings are relevant to guide future infection prevention and control strategies.

## Data Summary

The whole-genome sequences of the isolates from this study have been deposited at the European Nucleotide Archive (ENA) under the BioProject PRJEB52268.

**Impact Statement**

The zoonotic pathogen *Campylobacter jejuni* is among the leading causes of foodborne diseases globally and poses a public-health threat. To monitor and limit the transmission from animal to human, we need to understand how it evolves and acquires virulence and antimicrobial resistance (AMR) genes. We combined whole-genome sequencing and antimicrobial-susceptibility testing to investigate the population structure and genetic mechanisms of virulence and AMR for a large number of *C. jejuni* strains isolated over a period of 18 years. We revealed an increasing prevalence of resistance to quinolones and tetracycline over time, associated with *gyrA* mutation and the *tet(O*) gene, respectively. Finally, investigation of source attribution based on previous observations suggests that human infections are most likely related to isolates from poultry or generalist backgrounds, rather than isolates from ruminants or wild birds.

## Introduction

Campylobacteriosis is a leading cause of foodborne bacterial disease and the most reported zoonosis in Europe and North America [1, 2]. *Campylobacter* spp. show a broad animal reservoir, with poultry, ruminants and pigs being the predominant hosts [3]. Transmission of *Campylobacter* to humans occurs mainly through the consumption of contaminated animal products or cross-contaminated ready-to-eat products [4]. In humans, *Campylobacter jejuni* and *Campylobacter coli* are frequently isolated from patients with bacterial gastroenteritis.

Human cases of campylobacteriosis follow a clear seasonality, with most cases being reported during the summer months (June–August, in the northern hemisphere) [2, 5, 6]. This phenomenon is partly explained by the higher prevalence of *Campylobacter* in chicken broilers over this period of the year and the increased risk of poor food hygiene during the barbecue season [7]. Interestingly, a second peak of human campylobacteriosis has been recently reported in different countries, including Switzerland and Germany, during the winter holidays [2, 8, 9]. In this context, an association with consumption of specific meals (such as meat fondue, raclette grill meal and chicken liver products) has been proposed [10–12]. Increased travel during the holiday seasons in summer and winter may also foster campylobacteriosis transmission, with travelling abroad during Christmas and New Year having been found to be associated with almost three-time higher odds for contracting campylobacteriosis in Switzerland [8]. Therefore, the investigation of the circulating strains in Switzerland, their re-occurrence and similarities with publicly available strains of animal origin could be used to infer crucial information on possible transmission modalities.

Such investigations of circulating strains are also likely to provide additional insights into the ecology and evolution of *C. jejuni *, including its ability to resist antimicrobials. Antimicrobial resistance (AMR) is emerging globally among clinical *Campylobacter* isolates and has been recognized by the World Health Organization (WHO) as a problem of public-health importance [4, 13]. Various AMR-associated genes in bacteria are associated with horizontal gene transfer mechanisms, such as plasmids, which facilitate resistance dissemination. In *Campylobacter *, the most frequently encountered plasmids are the pTet and pVir plasmids, which harbour relevant genes associated with AMR and virulence. These include the *tet(O*) gene, which confers tetracycline resistance, and genes homologous to the type IV secretion system found in *Helicobacter pylori* [14–16]. In addition, the relationship between AMR and spontaneous mutations, mainly SNPs, has been reported in *Campylobacter* spp., resulting in clonal expansion of resistant lineages [13]. The genetic basis and dynamics of AMR in *C. jejuni* are not fully understood. We aimed to explore the distribution of AMR-associated genes and their persistence over time in a large collection of Swiss clinical isolates.

Besides gastroenteritis, infection with *Campylobacter* spp. is associated with Guillain–Barré syndrome (GBS), an acute polyneuropathic disorder [17, 18]. In *C. jejuni *, the presence of sialylated lipooligosaccharide (LOS^SIAL^)-related genes encoding β-1,3-glycosyltransferases, such as *wlaN* and *cgtB*, correlates with the ability to trigger GBS in patients suffering from campylobacteriosis. These genes are thought to be responsible for the molecular mimicry between the LOS^SIAL^ and the saccharide component of the human GM1 ganglioside present in human peripheral nerves [19]. Although previous studies have suggested a differential host-related distribution of the *wlaN* and *cgtB* genes in isolates from humans, broilers, and wild birds [20], additional studies are needed to confirm these findings.

In the last two decades, multilocus sequence typing (MLST) has been the method of choice to investigate the molecular epidemiology of *Campylobacter* spp. [21, 22]. However, whole-genome sequencing (WGS) has boosted resolution and has become the new reference standard for typing. The additional information provided by WGS-generated data is also improving our understanding of *Campylobacter* ecology, epidemiology and evolution, in particular the transmission of virulence factors and AMR determinants [23–25]. However, there are still several aspects to be clarified, including the routes of animal-to-human transmission of *Campylobacter *. For example, while previous source attributions studies revealed that certain *Campylobacter* lineages, or ecotypes, are preferentially isolated from specific hosts, others appear to be more adaptive and recently emerged as generalists that can infect multiple hosts [26, 27]. The WGS-based investigation of strains from different hosts and geographical areas is likely to provide additional insights into the routes of transmission, while also providing important information on the mechanisms of virulence and AMR present in these bacteria.

To elucidate these questions, here we combine WGS and antimicrobial-susceptibility testing (AST) to investigate the population structure and genetic mechanisms of virulence and AMR among 340 *C *. *jejuni* isolates from human patients with gastroenteritis in Switzerland over a period of 18 years. We characterize the main sequence types (STs) and most common clonal complexes (CCs), and show that the prevalence of resistance to quinolones and tetracycline in *C. jejuni* is increasing over time, likely due to clonal expansion of resistant determinants such as *gyrA* mutations and acquisition of the *tet(O*) gene. Our findings also suggest that human infections are most likely related to isolates from poultry or generalist backgrounds, rather than isolates from ruminants or wild birds.

## Methods

### Bacterial strains and culture conditions

A total of 340 *C *. *jejuni* strains isolated from stool samples from patients with gastroenteritis were randomly selected from the collection of the Swiss National Centre for Enteropathogenic Bacteria and Listeria (NENT). The collection covers all geographical regions of Switzerland and a timeframe between 2003 and 2020. The time of isolation was available for strains isolated after 2009 and the seasonality of major STs and CCs was investigated by comparing the number of strains isolated during different seasons. Seasons were defined as follows: spring (March–May), summer (June–August), autumn (September–November) and winter (December–February). Strains were stored at –80 °C in tryptone soy medium containing 30 % glycerol prior to recovery on Columbia blood agar containing sheep blood (Thermo Fisher) and incubated in a microaerophilic atmosphere at 42 °C for 24 h. A total of 3 % of the strains could not be recovered and were substituted by additional randomly selected strains. Data were fully anonymized and no information regarding the geographical origin nor patient records were available.

### Antimicrobial susceptibility testing

Phenotypic AST of *C. jejuni* isolates was performed using the microdilution method in cation-adjusted Mueller–Hinton broth with 5  % lysed horse blood, and minimum inhibitory concentrations (MICs) for erythromycin, ciprofloxacin, tetracycline, gentamicin, nalidixic acid and streptomycin (Sensititre, EUCAMP2; TREK Diagnostic Systems) were determined according to the European Committee on Antimicrobial Susceptibility Testing (EUCAST; version 12.0; www.eucast.org). Strain *C. jejuni* ATCC 33560 was used as a quality control, according to the manufacturer’s instructions.

### DNA sequencing

DNA extraction was performed on a Qiacube using the QIAamp DNA mini kit (Qiagen). Subsequently, DNA quantity was determined using a Qubit Fluorometer (Thermo Fisher) and quality was assessed using a Bioanalyzer TapeStation (D5000 ScreenTape; Agilent). Library construction was performed using NexteraXT or Nextera DNA Flex library kits (Illumina) and the constructed libraries were sequenced using NovaSeq 6000 (Functional Genomics Center Zurich), MiSeq (University Hospital Basel) or MiniSeq (Institute for Food Safety and Hygiene, University of Zurich) instruments (Illumina). All generated data were deposited at the European Nucleotide Archive (ENA) (BioProject PRJEB52268). In addition, libraries of strain N18-1277 prepared using the SQK-LSK109 kit were sequenced with MinION on a FLO-MIN106 flow cell (Oxford Nanopore Technologies).

### Genomic data quality analysis

Data quality control and assembly were performed using an established pipeline [28]. Briefly, raw reads were trimmed using Trimmomatic (version 0.39) with default parameters settings [29]. Assemblies were generated by unicycler (v0.3.0b) [30], and checked for standard quality parameters using quast (version 5.0.2) [31]. Genomes that met all the following quality criteria were included in the downstream analyses: estimated mean read coverage cut-off of ≥30×, 1.6 Mb ±160 kb genome assemblies, and contig count ≤200. The mean assembly length was 1 642 135 bp, with a mean read coverage of 98.2-fold, ranging from 30.2- to 269.4-fold. The mean G+C content was 30. 85 mol%, ranging from 30.06 to 31.13 mol%. A long-read assembly of strain N18-1277 was generated using Flye 2.8.1 [32], long-read polished with Medaka v1.5.0 (https://github.com/nanoporetech/medaka) and short-read polished with Polypolish v0.5.0 [33] and polca implemented in MaSuRCA v4.0.4 [34].

### Genotypic prediction of antimicrobial resistance

The identification of putative determinants conferring resistance to quinolones, erythromycin, aminoglycosides and tetracycline was performed as previously described [35]. Briefly, assembled contigs were screened for AMR-associated genes with ABRicate (version 0.8.10; https://github.com/tseemann/abricate) using the National Center for Biotechnology Information (NCBI) database [36], ResFinder 3.0 [37] and the Comprehensive Antibiotic Resistance Database (CARD) v3.1.0 [38], with a threshold for the identification of acquired genes of 90 % identity and 60 % minimum length. Chromosomal resistance-mediating point mutations were identified using the PointFinder database embedded in ResFinder 4.0 [39].

### Core- and pan-genome analysis

STs were deduced from the genome assemblies using mlst (v2.16.1; https://github.com/tseemann/mlst) and assigned to pre-defined CCs on PubMLST [40] (https://pubmlst.org/campylobacter). The same database was used to calculate core-genome MLST (cgMLST) (Oxford scheme) on PubMLST [40]. The relationship between STs, phenotypic resistance, seasonality and the presence of pTet plasmid was assessed using Fisher’s exact test (GraphPad Prism 9.1.2). Source attribution patterns were assigned based on ST–ecotype associations described in recent publications [41–43]. Gene annotation was carried out from the draft assemblies using Prokka v.1.13 [44]. The presence of plasmids or prophages was inferred from the assemblies using MOB-suite v.2.0.1 [45]. The predicted plasmid sequences were used as queries in blastn with threshold values set to >80 % coverage and >95 % identity. The sequences of accession numbers CP017866 and CP014746 were used to define the predicted sequences as pTet and pVir, respectively. Pan-genome analyses were carried out using Roary v.3.12.0 [46] with an amino acid identity cut-off of 95 % and splitting homologous groups containing paralogues into groups of true orthologues. A summary of the pan-genome composition and visualization of gene diversity is provided in Fig. S1(a–c), available with the online version of this article.

In parallel, whole-genome SNP (wgSNP)-based alignments were built from trimmed reads using the Snippy v.4.3.6 pipeline (https://github.com/tseemann/snippy). The closed genome of strain *C. jejuni* subsp. *jejuni* NCTC 11168 (GenBank assembly accession no. GCA_000009085.1) was used as a reference in read mapping. Areas of putative recombination were removed from the resulting alignment using Gubbins v.2.2.0 [47] and default settings (five iterations and >3 base substitutions to identify a recombination event). Maximum-likelihood phylogenies were obtained from the recombination-removed alignments using the tree building option FastTree v2.1.4 [48]. The core-genome phylogeny was visualized using iTOL [49] and the pan-genome genes calculated in Roary were displayed alongside the recombination-removed phylogenetic tree using Phandango [50] (https://jameshadfield.github.io/phandango). Virulence gene detection was carried out using ABRicate (version 0.8.10; https://github.com/tseemann/abricate) equipped with VFDB (Virulence Factor Database) [51]. Hits with less than 80 % identity or coverage were filtered out of the analysis.

The PubMLST *C. jejuni* database was screened for the major flagellin protein, FlaA (encoded by the *flaA* gene). All 2058 deposited *C. jejuni* sequences classified as CC-257 (as of November 4th 2022) were searched for the presence of the *flaA* sequence by blastn [NCBI, National Institutes of Health (NIH)] analysis using the DNA sequence from *C. jejuni* strain NCTC 11168 as reference. Presence of the gene was determined by >90 % alignment and identity with the query sequence (*E* value=0). Finally, the presence of type VI secretion system (T6SS) genes, encoding a total of 13 core components (TagH, TssA–TssG, TssI–TssM), was assessed using previously published reference sequences [52] and the blastn tool. The presence of genes was defined as DNA identity and coverage of ≥90 %.

### Time-scaled phylogenetic analysis

The trimmed reads of all genomes from two of the dominant groups, namely ST-21 and ST-50, were mapped using Snippy to related complete reference genomes obtained from public repositories: strain CAMSA2002 (NCBI accession no. GCF_017352015.1) and strain NCTC 12658 (NCBI accession no. GCF_019754215.1) were chosen for ST-21 and ST-50, respectively. Pairwise SNP distance matrixes from core-genome alignments were computed using snp-dists (version 0.8.2; https://github.com/tseemann/snp-dists) and were used to draw heatmaps combined with the corresponding dendrograms using the pheatmap package (https://github.com/raivokolde/pheatmap). One major cluster within each ST was identified based on SNP-number differences and using the recombination-free phylogenies generated by Gubbins for each sequence cluster, the significance of the temporal signal based on random permutations of sampling dates was assessed (BactDating; version.1.1) [53]. Root-to-tip linear regression analysis and calculation of the coefficient of determination (R^2^) was performed to infer the temporal signal of selected clusters within two dominant ST groups. Time-dated trees were estimated in a Bayesian inference framework in order to determine the date of all nodes of the trees.

## Results

### Multilocus sequence typing

**Fig. 1.**
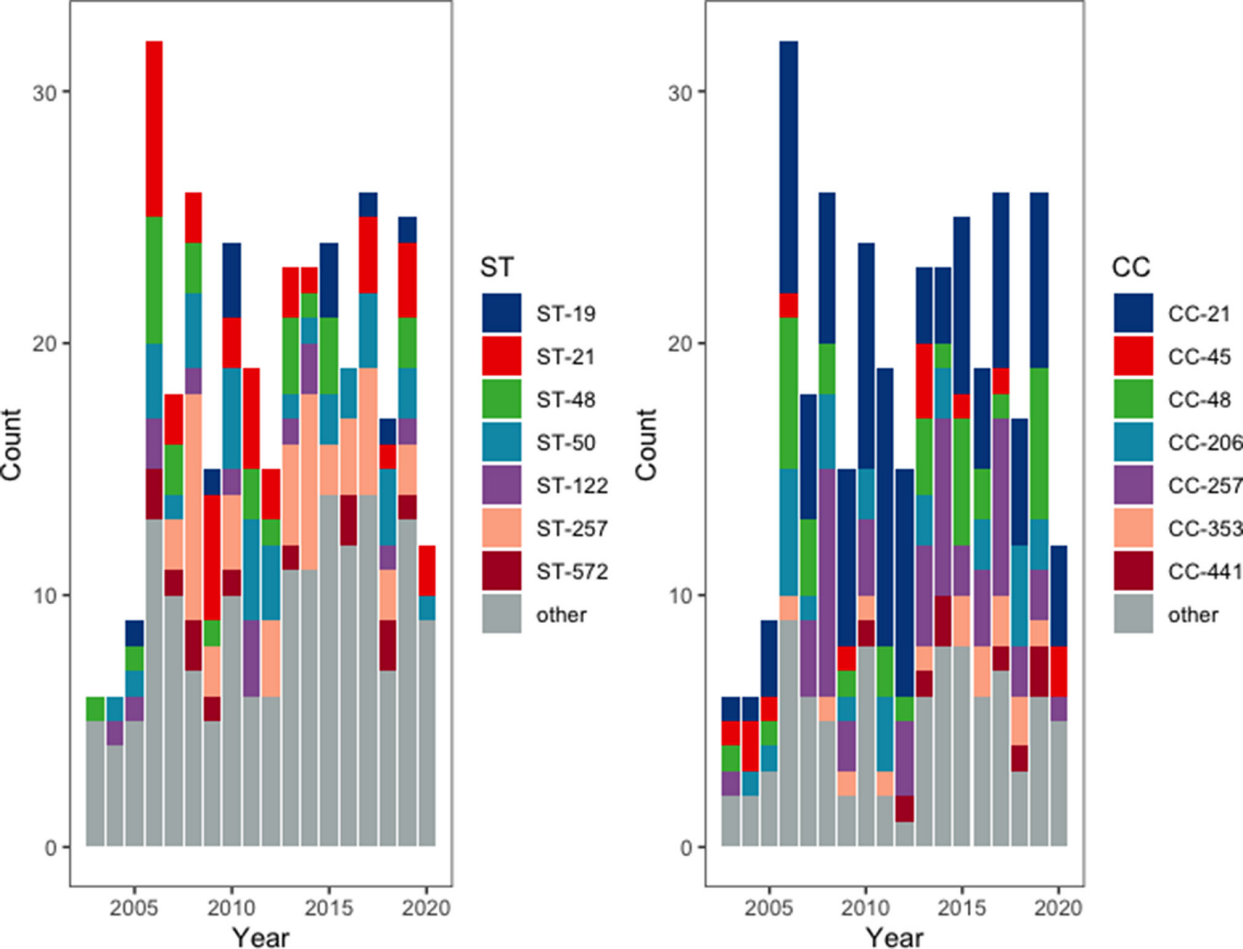
Stacked bar chart indicating the distribution of (**a**) STs and (**b**) CCs of 340 isolates from the Swiss NENT between 2003 and 2020. Major STs and CCs (>10 isolates) are shown individually, less frequent isolates are grouped as other.

We sequenced 340 genomes of *C. jejuni* isolated from human patients with gastroenteritis in Switzerland between 2003 and 2020. Multi-locus STs were extracted from all 340 assemblies and showed a heterogenous dataset. A total of 68 unique STs appertaining to 22 CCs were assigned to 304 sequenced strains, including 5 novel STs (which have been submitted to pubmlst.org). For the remaining 36 samples, an ST could not be determined because one or more loci were missing or incomplete. The most common MLST types from this collection of human isolates were ST-257 (*n*=44), ST-21 (*n*=36) and ST-50 (*n*=35), whereas the most common CCs were CC-21 (*n*=102), CC-257 (*n*=49) and CC-48 (*n*=33) (Fig. 1). Overall, more strains were isolated during the summer months (*n*=85) compared to the winter months (*n*=40). The prevalence of ST-257 was significantly higher during the winter (32.5 %) compared to the summer (5.8 %; *P*=0.0002). For the remaining major STs and CCs, no significant correlation with seasonality was observed.

### Core-genome analysis

**Fig. 2.**
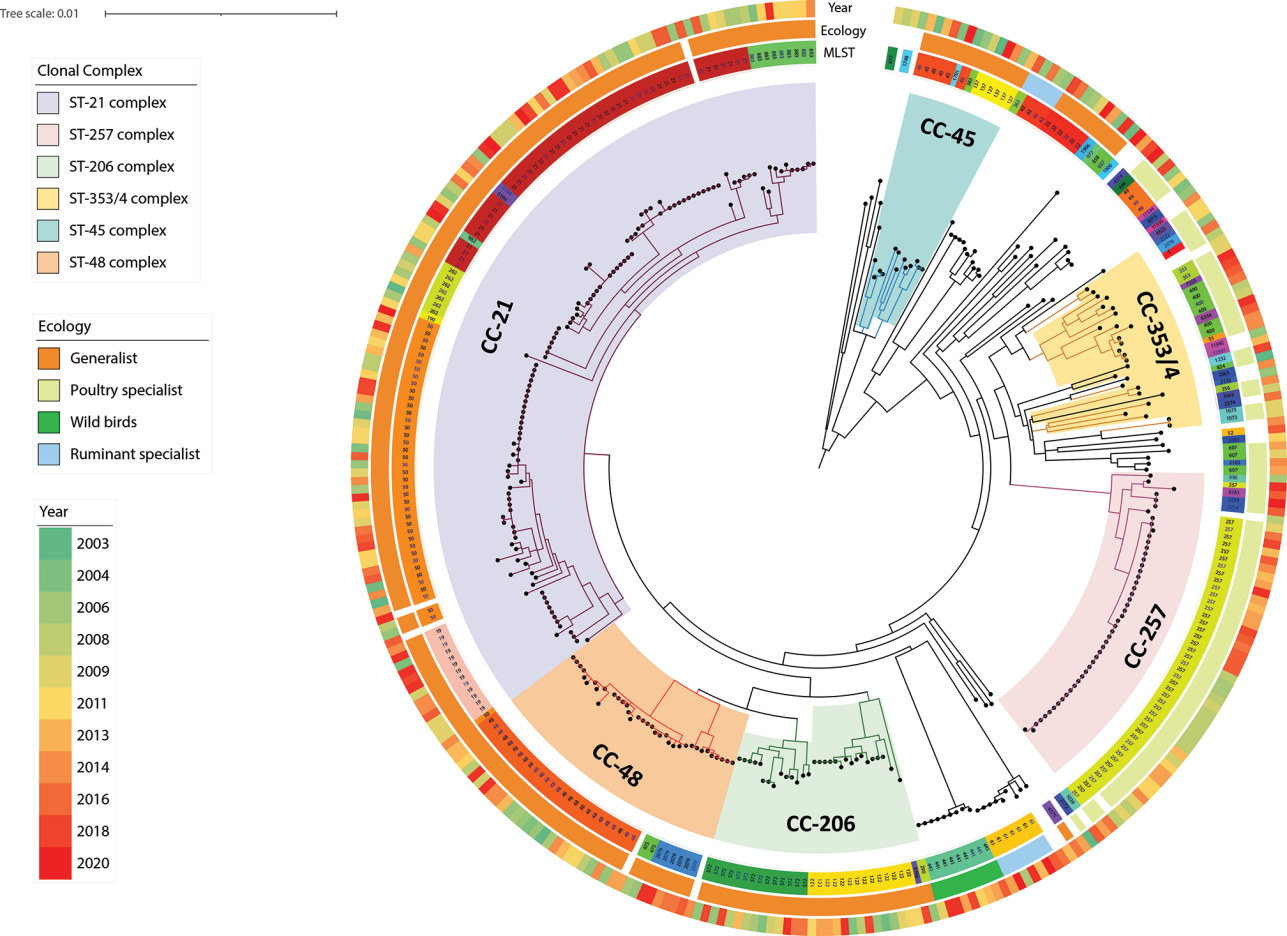
Population structure of *C. jejuni* based on cgMLST with STs and CCs colour-coded in the inner ring (ST numbers are labelled on the coloured segments) and on the tree, respectively. Source attribution of the different STs and the year of isolation are shown in the second ring and in the outer ring. The scale bar is expressed as number of cgMLST allele differences between isolates.

A total of 966 core genes were present in ≥95 % of the *C. jejuni* genomes and a set of 4894 additional genes were identified in at least one of the genomes under consideration (Fig. S2). The core genes were used to derive the *C. jejuni* population structure using cgMLST. The resulting structure supports the stable presence of STs such as ST-21, ST-50, ST-48 and ST-257 over the years (2003–2020) (Fig. 2). At the same time, more distantly related strains with unique or rare STs, such as ST-977 and ST-4823, were only present sporadically.

Three major groups, namely CC-21 (which includes ST-21 and ST-50), CC-48 and CC-206 appear to be phylogenetically related, whereas isolates belonging to CC-45, CC-353 and CC-354 showed a greater genetic distance between sequences from the same CCs and different CCs (Fig. 2). Interestingly, isolates from CC-257, in particular those from ST-257, were highly similar even though persistence over the entire study period was observed (Fig. 2).

Source attribution investigation based on CCs assigned 188 isolates to the generalist category (55.3 %), 83 as poultry specialist (24.4 %), 11 as ruminant specialist (3.2 %) and 9 as wild bird origin (2.7 %). Notably, the phylogenetic distribution obtained using cgMLST typing successfully split major groups and their source attribution: strains from CC-21, CC-48 and CC-206 grouped in the phylogeny were classified as generalist; whereas poultry specialist strains, encompassing CC-257, CC-353, CC-354 and other STs detected less frequently, shared a common internal node on the tree (Fig. 2).

### Time-scaled phylogenetic analysis

**Fig. 3.**
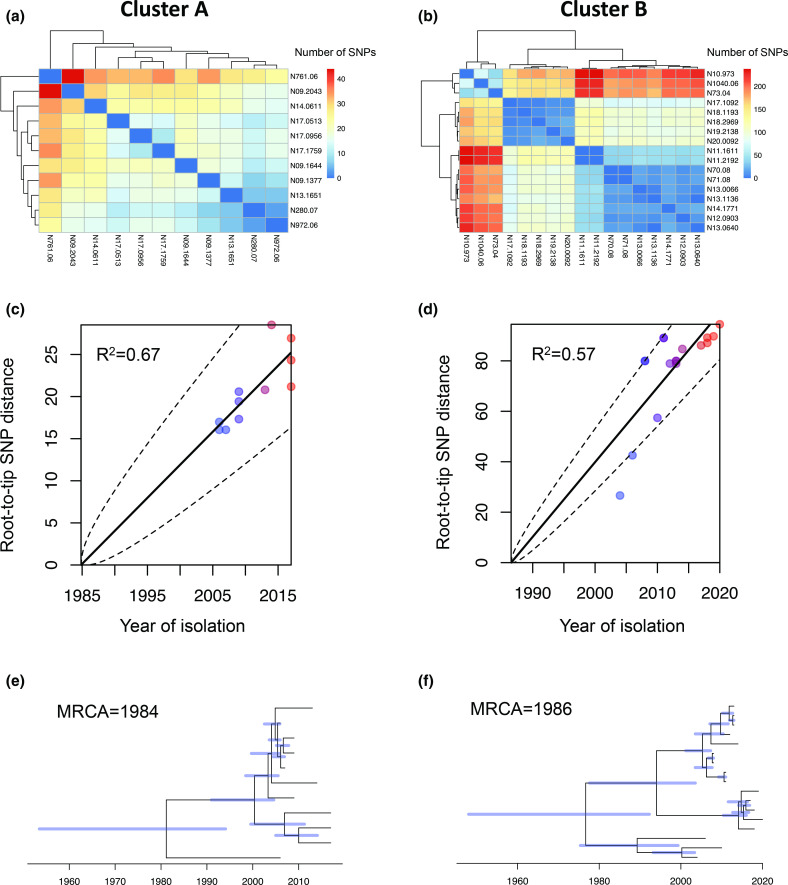
Heatmaps of pairwise comparison matrix derived from SNP distances combined with the corresponding dendrograms for cluster A (**a**) and cluster B (**b**). Blue to red colours indicate the increasing number of SNPs between sequenced strains. Linear regression of modelled root-to-tip SNP distances for cluster A (**c**) and cluster B (**d**). The colours of dots on the tips of branches represent the date of isolation, with red depicting recent isolates and blue older isolates. Bayesian maximum clade credibility time-calibrated phylogenies based on recombination-free regions of the core genome of cluster A (**e**) and cluster B (**f**). The most recent common ancestors (MRCAs) were predicted at approximately the year 1984 (range 1955–1995) and year 1986 (range 1950–1990) for cluster A and cluster B, respectively. Blue horizontal bars at each node represent 95 % confidence intervals.

We then investigate in more detail the evolutionary history of the strains belonging to the most dominant CC, CC-21. Analysis of SNP distances within this group revealed over 7000 and 5000 SNP differences between the most distant strains from the ST-21 and ST-50, respectively (Fig. S3). One major cluster within each of those STs was identified based on SNP-number differences: cluster A (ST-21), comprising 11 isolates obtained between 2006 and 2017; and cluster B (ST-50), comprising 17 isolates obtained between 2004 and 2020. These clusters were chosen for time-scaled phylogenetic analysis. A maximum of 44 SNPs between sequences in cluster A and 238 SNPs between sequences in cluster B were detected (Fig. 3a, b). Although the number of sequences included in the time-scaled analysis was small, a significant positive correlation between the dates of isolation and root-to-tip SNP distances for both clusters was observed (R^2^=0.67 and *P* ≤2×10^−4^ in cluster A; R^2^=0.57 and *P* <1×10^−4^ in cluster B), indicating the presence of a clock-like signal (Fig. 3c, d). Under clock-like evolution, we expected a linear relationship between the sampling year and the number of nucleotide substitutions along the tree [54]. The most recent common ancestors were predicted at approximately the year 1984 (range 1955–1995) for cluster A and year 1986 (range 1950–1990) for cluster B (Fig. 3e, f). Weak correlation between the dates of isolation and root-to-tip distances was observed after including more distantly related strains in the time-scaled analysis (data not shown).

### Virulence genes

**Fig. 4.**
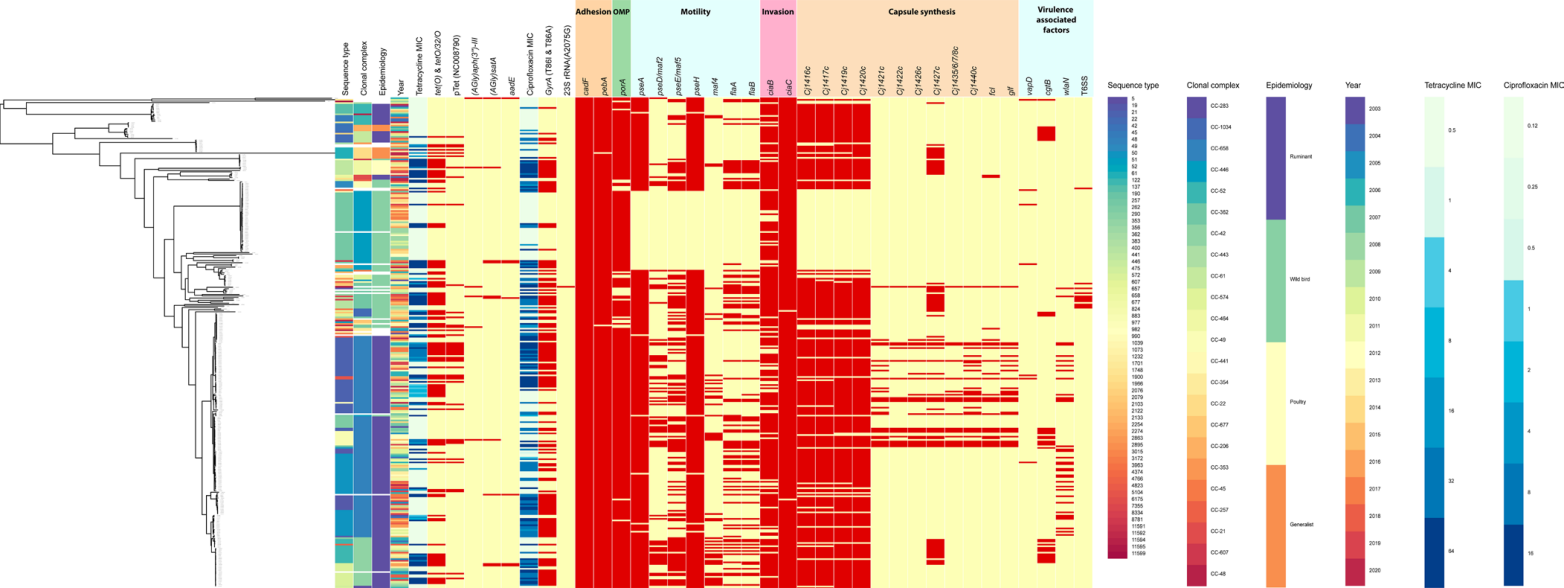
Maximum-likelihood pan-genome-based phylogeny and gene distribution map from annotated assemblies. The phenotypic and genotypic prediction of resistance against clinically relevant antimicrobials and the correlation with the pTet plasmid is displayed. Suggested source attribution of the different STs, the year of isolation and the detection of major virulence-associated genes are shown.

The full genomes of the 340 isolates were also used to assess the presence of known virulence genes associated with *Campylobacter* adhesion, invasion, motility, toxin and secretion system. Genes encoding outer membrane proteins (*cadF*, *pebA*, *porA*) and invasion proteins (*ciaBC*) were present in most isolates (80–100 %), whereas motility-associated genes (*flaAB*, *pseD/maf2*) and genes associated with capsule synthesis (*Cj1421c*, *Cj1422c*, *Cj1426c*, *Cj1427c*, *Cj1435c*, *Cj1436c*, *Cj1437c*, *Cj1440c*, *fcl*, *glf*) were detected with lower frequencies (5–25 % in CC-21 or CC-48) or sometimes even completely absent (such as in ST-257). Only 105 (5 %) out of 2058 deposited *C. jejuni* sequences in PubMLST and classified as CC-257 presented the motility-associated gene *flaA* and only a few of them were ST-257. Despite the lower frequency of capsule synthesis genes overall, we were able to detect some specific clusters across the panel of isolates – e.g. *Cj1427*, encoding an enzyme responsible for catalysing the oxidation of GDP-d-glycerol-α-d-manno-heptose, was present in 100 % (9/9) of ST-441 isolates [55]. The *wlaN* and *cgtB* genes, both associated with GBS, showed a differential distribution across STs. In particular, 36–40 % of the isolates from ST-19 (*n*=11) and ST-50 (*n*=35) carried the *wlaN* gene, and 78–83 % from ST-122 (*n*=11) and ST-22 (*n*=6) carried *cgtB*. In comparison, none of the isolates from certain CCs, such as CC-257 (*n*=49) and CC-48 (*n*=33), carried these relevant genes (Fig. 4). None of the isolates possessed both *wlaN* and *cgtB* genes, and there was no relationship between the presence of GBS-associated genes and the year of isolation, with both genes being detected over the entire study period. In terms of distribution of these genes and the source attribution, the large majority of the isolates presenting *wlaN* and *cgtB* were classified as generalists. Poultry specialists showed low frequencies (2.4–3.6 %) of GBS-associated genes, with two isolates (both CC-353) that were *wlaN* positive and three isolates (CC-607) that were *cgtB* positive being the only exceptions. Ruminant specialists presented *cgtB* only (27.3 %) and none of the wild-bird-associated isolates carried GBS-associated genes. Overall, low prevalence (3 %, *n*=11) of a complete T6SS was observed. Except for one isolate, the remaining isolates carrying all 13 genes associated with T6SS were from CC-353 (Fig. 4). Interestingly, six T6SS-negative *C. jejuni* strains were found to possess an incomplete system with only 5 out of 13 genes (*tssA–tssF*). Furthermore, the genome of the *C. jejuni* N15-0246 strain was identified as the only one without a complete T6SS cluster to present the *tssD* (*hcp*) gene.

### Phenotypic antimicrobial susceptibility testing

To complement our genomic analyses, we performed AST to determine the MICs for erythromycin, ciprofloxacin, tetracycline, gentamicin, nalidixic acid and streptomycin of all *C *. *jejuni* isolates. A total of 140 (41.1 %) isolates were pan-susceptible to all antimicrobials tested (Table S1). The highest frequency of resistance was observed for quinolones (49.8 %), followed by tetracycline (36.9 %) and erythromycin (0.3 %). Gentamicin resistance was not observed among the tested strains.

**Fig. 5.**
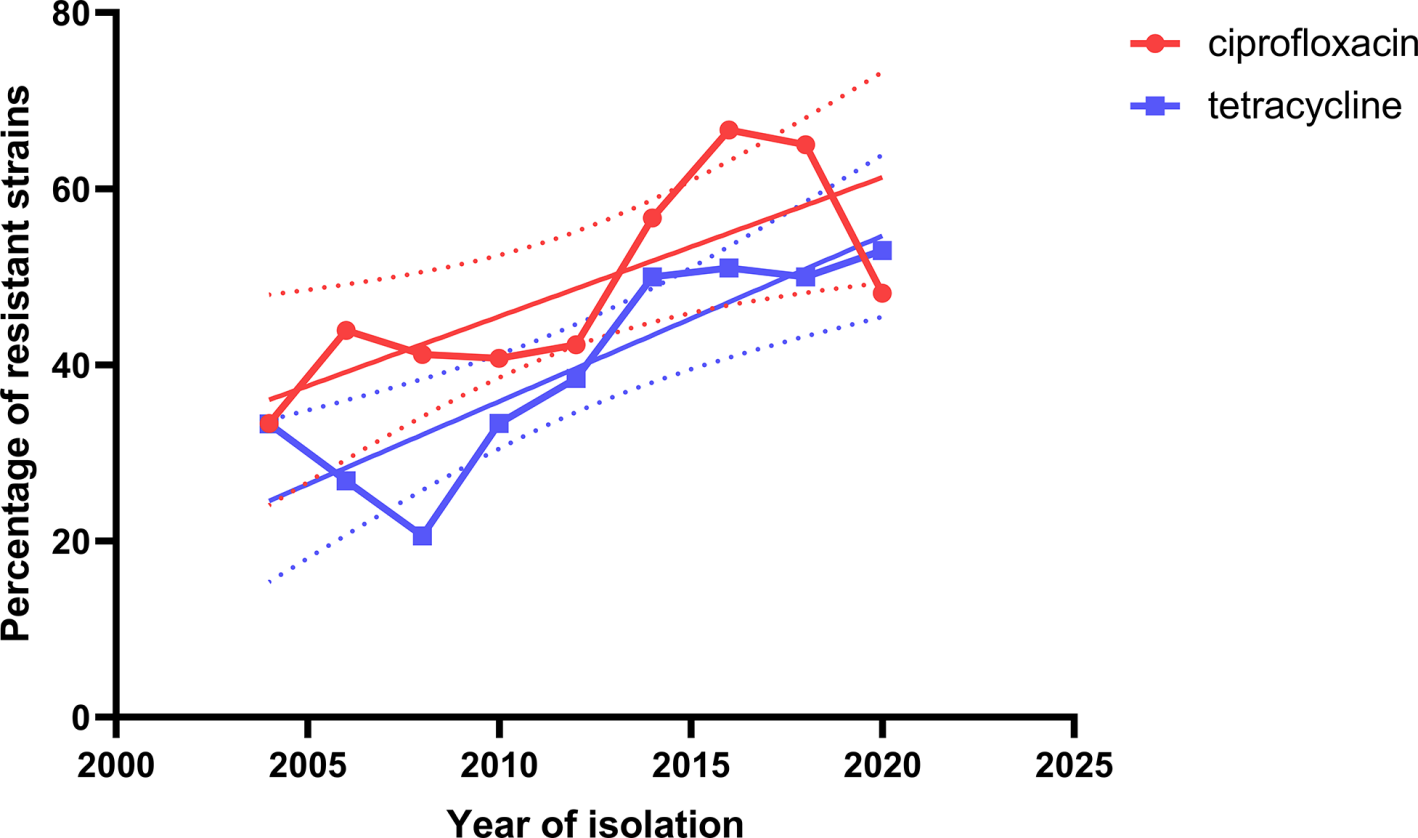
Increase in AMR rates against ciprofloxacin and tetracycline over the study period (2003–2020). Linear regressions indicating the trend of AMR over time and confidence intervals (dotted lines) are shown.

Notably, we found an increased frequency of AMR from 2004 to 2020, particularly for ciprofloxacin (47.5–59.5 %) and tetracycline (35.4 –45.8 %) (Fig. 5). We observed that MIC values and, consequently, resistant isolates were unequally distributed among STs (Fig. 4). Strains from ST-572 (*P*<0.001) and ST-19 (*P*=0.0112) were significantly more resistant to ciprofloxacin and nalidixic acid than strains from other STs. ST-21 presented significantly more strains resistant to tetracycline (*P*=0.0131). Conversely, lower quinolone and tetracycline MICs were observed for ST-257 (*P*<0.0001) strains compared to other STs.

### Resistome

We next screened the whole-genome sequences of all isolates in our collection to detect genes and point mutations potentially associated with resistance, such as chromosomally encoded antibiotic-resistance modifications and genes harboured on plasmids. Phenotypic and genotypic AMR results were concordant in 323 out of 340 isolates (95 %), with 94.1 % ciprofloxacin resistant and 94.4 % tetracycline resistant phenotypes correctly predicted (Fig. 4). Conversely, 98.8 % of the ciprofloxacin-susceptible and 99.5 % of the tetracycline-susceptible phenotypes were correctly predicted.

In quinolone-resistant isolates, we identified chromosomal *gyrA* mutations T86I (99.4 %) and T86A (0.6%) (Table S1). In tetracycline-resistant isolates, *tet(O*) (79.8 %) and mosaic *tet* gene (specifically *tetO/32/O*, 20.2 %) were detected. These genes related to tetracycline resistance were found both encoded in the chromosome (60 %) or in plasmids (40 %; pTet GenBank accession no. NC008790). A small number of isolates (2.6 %) was able to grow in the presence of the first-class aminoglycoside streptomycin (MIC ≥16 µg ml^−1^). These streptomycin-resistant isolates all harboured associated resistance elements, such as aminoglycoside 6-adenylyltransferase [*aadE* or *aad(6*)], ANT(6)-I aminoglycoside *O*-nucleotidyltransferases or ribosomal RPSL protein. Additional aminoglycoside-resistance genes [1.8 % *aph(3')-III*; 2.9 % *satA*] were detected in streptomycin-resistant isolates. We also found a single isolate phenotypically resistant to erythromycin (MIC=64 µg ml^−1^), which contained a SNP in the 23S rRNA gene (at A2075G) known to be linked to macrolide resistance.

**Fig. 6.**
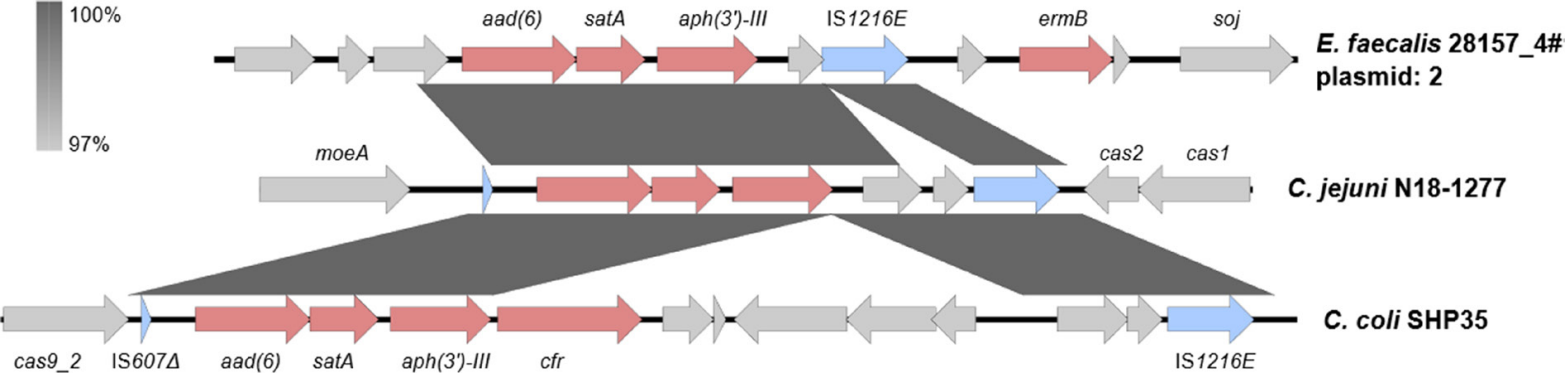
Genetic context of the region harbouring the *aad(6*), *satA* and *aph(3')-III* genes in *C. jejuni* N18-1277. The region was compared to similar resistance gene cassettes identified in publicly available sequences of *E. faecalis* 28157_4# plasmid 2 (LR962354.1) and of the chromosome of *C. coli* SHP35 (MF037586.1). Shaded boxes between sequences indicate homologous regions (>95 % sequence identity). Resistance genes (red) and intact or partial transposases (blue) are coloured. The figure was generated with Easyfig 2.1 [79].

We also investigated the presence of chromosomally encoded AMR genes associated with mobile elements, which could be associated with extensive horizontal gene transfer events leading to the relatively large accessory genome detected (Fig. S2). We detected a novel chromosomal cassette in strain N18-1277 carrying several resistance genes, including *aph(3')-III*, *satA* and *aad(6)*, and flanked by insertion sequence elements (Fig. 6). All resistance genes on this cassette showed amino acid identities above 90 % to database references except for *aad(6)*, which showed an identity of 85 % to accession number AY712687 in CARD. Comparative genomic analysis of this region and nucleotide blast searches revealed high similarity to resistance gene cassettes identified in publicly available sequences (Fig. 6) of *Enterococcus faecalis* 28157_4# plasmid 2 (GenBank accession no. LR962354.1; 3216/3218, 99 % identity) and a region within the chromosome of *C. coli* SHP35 (GenBank accession no. MF037586.1; 2784/2868, 97 % identity).

Finally, we analysed the presence of resistance genes in plasmids. The pTet plasmid was found in 14.1 % (*n*=48) of the isolates, all of which displayed phenotypic resistance to tetracycline, likely via horizontal gene transfer of the *tet(O*) and *tet(O/32/O*) genes. The pTet plasmid was present in isolates belonging to 17 different STs and from every year of the study since 2004. Significant differences in pTet plasmid distribution were observed between isolates, with plasmid frequency being higher in strains belonging to CC-21 (24.4 %; *P*=0.0023) and CC-353 (25 %; *P*=0.0113) compared to CC-257 (2.1 %) (Fig. 4). In five isolates, the pTet plasmid carried the virulence-associated protein 2 (VapD) encoding gene. Various additional AMR-associated genes, including *aph(3')-III* (three isolates) and *satA* (two isolates), were also found on pTet plasmids. Strain N2703-03 was the only isolate found to contain a pVir plasmid encoding type IV secretion system homologous genes (*Cjp54*, *virB10*, *virB11*, *virB4*, *virB8*, *virB9*, *virD4*).

## Discussion

In this study, we performed WGS and AST on 340 *C *. *jejuni* isolates collected between 2003 and 2020 from patients with gastroenteritis from Switzerland. The observed population structure supports the stable presence of specific STs, such as ST-21, ST-50, ST-48 and ST-257, over the years. These findings suggest persistence of clinically relevant strains in farm animals or other sources of infection over time. At the same time, more distantly related strains with unique or rare STs, such as ST-977 and ST-4823, were only detected sporadically during the study period and were most likely associated with foreign travelling or occasional transmission events from uncommon animal or environmental sources. Three major groups, namely CC-21 (which includes ST-21 and ST-50), CC-48 and CC-206 appear to be phylogenetically related, whereas isolates belonging to CC-45, CC-353 and CC-354 showed a greater genetic distance between sequences from the same CCs and different CCs. Interestingly, isolates from CC-257 and, in particular, those from ST-257 were highly similar even though persistence over the entire study period was observed. Isolates from ST-257 showed significant differences in their seasonality and were isolated more often during the winter compared to the summer months.

We observed high frequencies of resistance for ciprofloxacin and nalidixic acid (49.8 %), followed by tetracycline (36.9 %). Similar results were reported from a recent monitoring programme investigating cecum samples from Swiss broilers and showing that 51.4 % of *C. jejuni* were resistant to quinolones and 40.0 % to tetracycline [35]. Interestingly, in our study, strains belonging to ST-257, described as a poultry specialist ST in previous reports [26, 41], presented significant lower quinolone and tetracycline MICs compared to strains from other STs. Conversely, our findings revealed that two major MLST complexes, CC-21 (ST-19–21) and CC-206 (ST-572), both showing a generalist behaviour, presented higher MIC values for quinolones and tetracycline, respectively. Similar patterns of resistance were observed by Cody *et al*. between 2003 and 2010, with CC-206 being associated with ciprofloxacin resistance in patients from the UK [56]. We also found that the majority of strains assigned to CC-45 were pan-susceptible to antimicrobials and showed a greater genetic distance compared to other CCs observed (Fig. 2), supporting the hypothesis that CC-45 might be a generalist that is also commonly found in the environment and can sporadically be found in humans [57, 58].

With regards to tetracycline resistance, a high percentage (36.9 %) of *C. jejuni* isolates carried the *tet(O*) gene, which encodes the ribosomal A site TetO binding protein. The pTet plasmid was found in 14.7 % of the isolates belonging to 17 different STs and from each year of the study since 2004. Significant differences in pTet plasmid distribution were observed, with higher prevalence (25 %) in isolates belonging to CC-21 and CC-353 compared to CC-257 (2.1 %). These data highly correlate with phenotypic testing and all strains carrying pTet were tetracycline resistant according to AST.

In terms of quinolone resistance, we observed mutations such as T86I, encoded within the quinolone-resistance-determining region (QRDR) of *gyrA*. Quinolone resistance was common in the Swiss strains analysed in this study (49.8 %), with a clear increasing trend over the study period, in line with observations from other countries [59]. A significant association between quinolone resistance in clinical *C. jejuni* and the administration of such antimicrobials in food-producing animals has been reported, which suggests the animal origin of such resistance [60].

Other target modifications of clinical concern were observed, including point mutations (e.g. A2075G) in the peptidyl encoding region in domain V of the 23S rRNA gene, which leads to high-level macrolide resistance [61]. This mutation was found in one *C. jejuni* Swiss strain resistant to erythromycin, which is one of the drugs of choice in human medicine nowadays.

We also evaluated the presence of streptomycin-resistance genes, finding members of the aminoglycosides adenyltransferases (AADE) family, such as the *aadE* gene, in approximatively 6 % of the strains isolated since 2016. These strains belonged predominantly to three CCs, namely CC-257, CC-206 and CC-353. In addition, one strain presented a mutation in the 30S ribosomal subunit (*rpsL*) that confers a high-level of streptomycin resistance in *Campylobacter* [62]. In accordance with previous observations [63], isolates carrying the *aadE* resistance gene exhibited additional AMR-associated genes or mutations, such as *gyrA* and *tet(O),* and four of these isolates shared a probable common ancestor.

We also report the identification of one strain (N18-1277) presenting a chromosomal cassette containing multiple resistance genes flanked by insertion sequences. Insertion sequences are mobile genetic elements commonly found in bacterial genomes and are known to favour horizontal transfer events and transposition from plasmids into the chromosome. The insertion sequence elements flanking AMR genes, such as those reported in strain N18-1277, might have played an important role in the chromosomal acquisition of the cassette, since similar elements have been reported in *C. jejuni* plasmids [64]. As for other genes horizontally transferred to and between *Campylobacter* sp., such as *tet*(O) and *erm*(B), the high homology of the chromosomal cassette to Gram-positive sequences, especially those originating from *E. faecalis *, suggests a Gram-positive source of aminoglycoside resistance genes, which are horizontally transferred to *Campylobacter* [65]. These findings suggest that increased surveillance and larger investigations addressing the occurrence of mobile elements that confer resistance to clinically relevant antibiotics in *Campylobacter* are needed to better understand the evolution of AMR in this pathogen.

We also observed high concordance between phenotypic and genotypic AST, e.g. 94.1 % of the ciprofloxacin-resistant isolates were correctly identified based on genetic determinants of resistance. However, we were unable to identify genes linked to antibiotic resistance in ten isolates phenotypically resistant to the drug, possibly due to AMR mutations not covered by the current databases. Notably, different factors may influence the lack of AMR genotype–phenotype correlation, including over- and under-expression of efflux pumps or porin mutations. While conventional diagnostics procedures including the culture of pure isolates and determination of MICs remain recommended to enable the detection of new resistance mechanisms, the high concordance between phenotypic and genotypic AST shown in the present study suggests that the implementation of short- and long-read sequencing for diagnostic purposes and determination of AMR represent a valid alternative to conventional methods.

Our data also provide insights into the evolution of some of the strains belonging to the most dominant *C. jejuni* CC in Switzerland. Analysis of SNP-distances within ST-21 and ST-50 groups was performed. Interestingly, some groups of strains isolated over several years are separated by relatively small numbers of SNPs, as is the case for the seven ST-21 strains in cluster A isolated between 2010 and 2016, which show a maximum of 32 SNP differences between the most distant strains within the cluster (Fig. S3). This suggests that certain *C. jejuni* strains reoccur in Swiss human patients over time, most likely being transmitted to humans after persisting in specific ecological niches. While the small number of isolates in clusters A and B prevent a confident dating of common ancestors in the present study, the significant temporal signal observed for these clusters reinforces the concept of persistence mentioned above.

Our results also provide novel information on the distribution of virulence genes in *C. jejuni* isolates from Swiss patients. Recent studies have demonstrated that single *Campylobacter* clades are associated with increased virulence and cause more severe gastrointestinal infections compared to less pathogenic strains found in asymptomatic patients [66, 67]. In our collection, strain N2703-03 was found to contain the pVir plasmid encoding genes (*Cjp54*, *virB10*, *virB11*, *virB4*, *virB8*, *virB9*, *virD4*) homologous to the type IV secretion system found in *H. pylori* [68]. A connection between the presence of this plasmid and bloody diarrhoea in human patients has been speculated; however, the role of pVir in the pathogenesis of invasive infections has not been confirmed [69]. We also detected the presence of LOS^SIAL^-related genes, namely *wlaN* and *cgtB*, encoding β-1,3-glycosyltransferase enzymes that have been correlated with the ability of *C. jejuni* to trigger GBS in patients suffering from campylobacteriosis in previous studies [20]. In our isolates, the presence of the LOS^SIAL^-related genes, and in particular the *wlaN* gene, showed a clonal distribution, which is in accordance with previous observations [20, 70]. In terms of source attribution, GBS-associated genes were mostly observed in isolates defined as generalists. Ruminant specialists presented the *cgtB* gene only and none of the wild-bird-associated isolates carried GBS-associated genes. This may indicate a host-related occurrence or absence of the enzymes required for the production of LOS^SIAL^. Interestingly, the presence of a complete T6SS was lineage-related and we could identify all T6SS core components in only 3 % of the sequences. Except for one isolate, all T6SS-positive sequences were from CC-353 and this observation correlates with previous publications [71, 72]. Finally, the detection of the gene *tssD* (*hcp*) as a proxy for determining the presence of a functional T6SS showed highly satisfactory results. Our analyses also included source attribution, to gain insights into the potential routes of transmission of *C. jejuni* to humans. Our findings confirm that major CCs, including CC-21, CC-48 and CC-206, which are believed to be host generalists and colonize a wide range of host species based on previous observations [73], are closely related phylogenetically. By contrast, we found other CCs that are mostly associated with environmental niches (ST-45) or with specific animal species (and therefore considered to be host-specific, such as CC-61) [41, 74–76], which showed a greater distance in the phylogeny, suggesting distinct evolutionary events. Our findings are also consistent with previous population genomics studies, which have shown that certain lineages of *C. jejuni* adapted to different hosts by downregulating virulence determinants or changing virulence gene repertoire [77, 78]. For example, we found that poultry-associated isolates, namely ST-257, do not harbour motility genes *flaAB* and *pseD/maf2* and genes associated with capsule synthesis, which likely reflects adaptations to this host. Notably, these isolates were genetically similar and reoccurred in human patients over a long time period. The factors that promote such adaptive behaviour and host switching are poorly understood, and the selection of beneficial elements in the pathogen is potentially influenced by external effects such as diet and breeding practices. The absence of known motility-associated genes in ST-257 is supported by the screening of all CC-257 genomic sequences available in PubMLST for the *flaA* gene, which was present in 5 % of 2059 sequences and only few of them were ST-257. While the source of infection for humans is often unknown, the handling, preparation and consumption of broiler meat are estimated to account for a large proportion of all clinical cases. Therefore, kitchen and food hygiene is crucial to avoid cross-contamination of food products and limit human infections. The identification of local host reservoirs is also critical for the understanding of transmission dynamics and the implementation of monitoring measures.

We are aware that our study is limited by our collection framework and the lack of metadata, such as information on the severity of the infection, consumption of specific meals prior to infection, and travel history of the patients. Unfortunately, this data was not submitted and is not available retrospectively.

### Conclusion

The present analysis of a large number of *C. jejuni* isolates from Swiss patients over an 18 year period revealed an increasing prevalence of resistance to quinolones and tetracycline over time, linked to clonal expansion of *gyrA* mutants and acquisition of the *tet(O*) gene, respectively. WGS was used to gain insights into *the C. jejuni* population, revealing high heterogeneity among the observed STs, with the most abundant ST recurring over the entire study period, while other STs were only observed sporadically. Investigation of source attribution based on previous observations suggests that human infections are most likely related to isolates from poultry or generalist backgrounds, rather than isolates from ruminants or wild birds.

## Supplementary Data

Supplementary material 1

Supplementary material 2

Supplementary material 3

Supplementary material 4
