# Supplementary material for: Whole-genome-based characterization of Campylobacter jejuni from human patients with gastroenteritis collected over an 18 year period reveals increasing prevalence of antimicrobial resistance
Source: Microb Genom. 2023 Feb 21;9(2):mgen000941. doi: 10.1099/mgen.0.000941 (PMC9997746; doi:10.1099/mgen.0.000941)
Supplement: Supplementary material 1 [file mgen-9-941-s001.pdf]

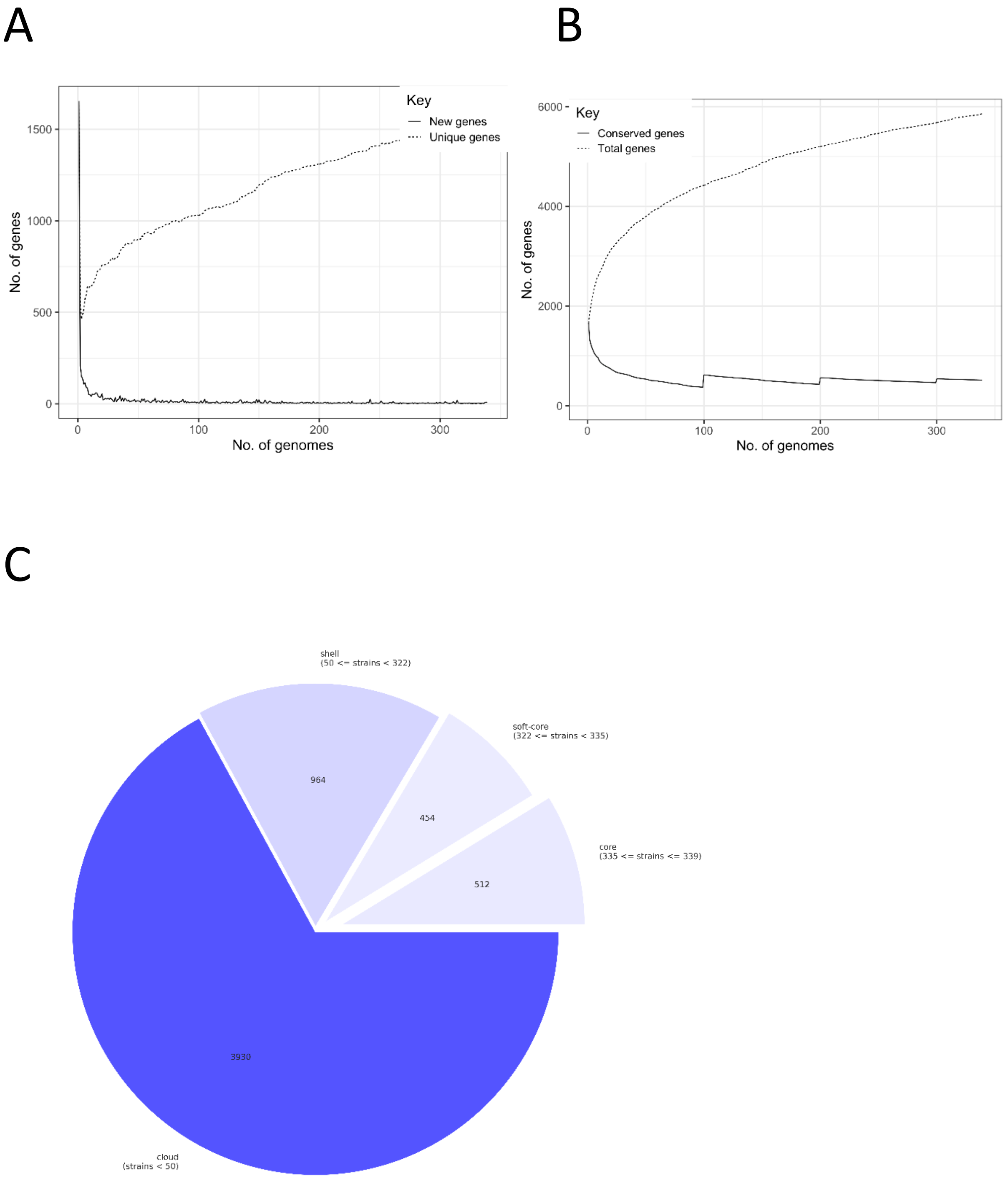

Supplementary Figure. 1. Visualization of gene diversity was achieved using the native Rscript from Roary (create_pan_genome_plots.R) (**A-B**). The summary of the pan-genome composition provided by Roary was visualized using the open-source python script 'roary_plots.py' (https://github.com/sanger-pathogens/Roary/tree/master/contrib/roary_plots) (**C**).
