# Supplementary material for: Whole-genome-based characterization of Campylobacter jejuni from human patients with gastroenteritis collected over an 18 year period reveals increasing prevalence of antimicrobial resistance
Source: Microb Genom. 2023 Feb 21;9(2):mgen000941. doi: 10.1099/mgen.0.000941 (PMC9997746; doi:10.1099/mgen.0.000941)
Supplement: Supplementary material 2 [file mgen-9-941-s002.pdf]

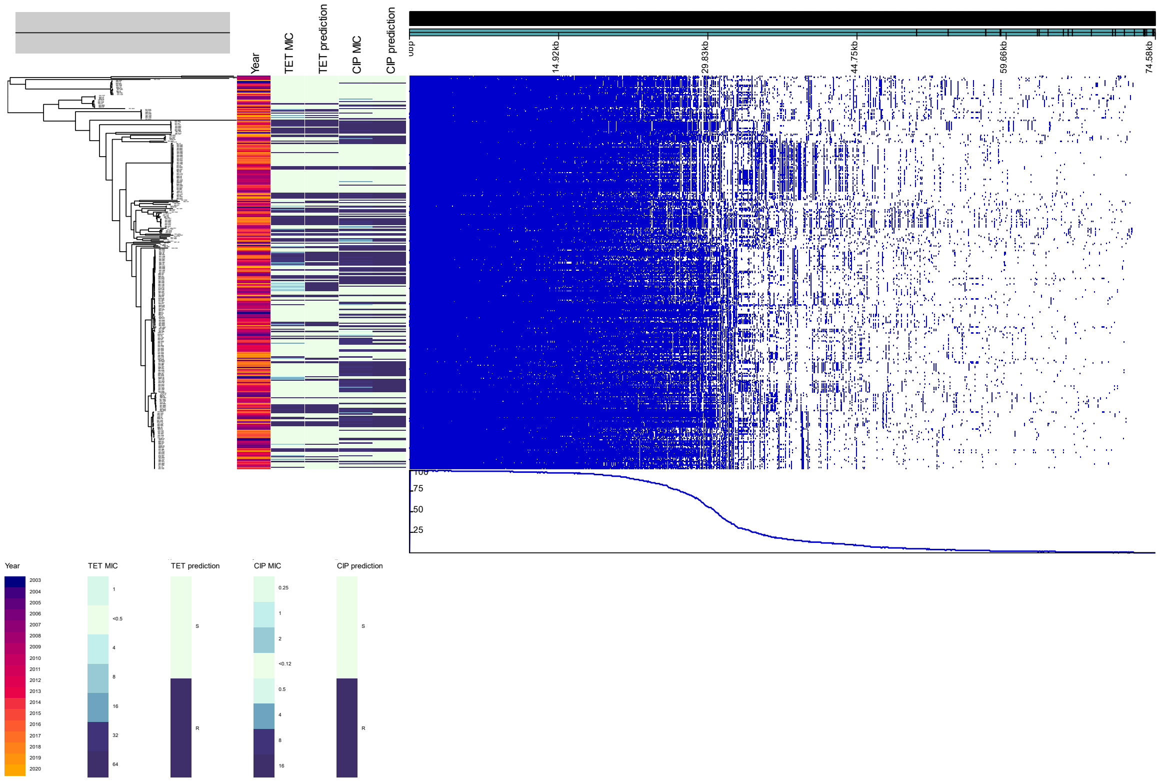

Supplementary Figure 2. Gene distribution map of *C. jejuni* pan-genome from annotated assemblies. On the left side of the figure is an unrooted, maximum likelihood pan-genome-based phylogeny. The year of isolation, the outcome of phenotypic antimicrobial susceptibility testing, genotypic prediction of resistance against tetracycline (TET), and ciprofloxacin (CIP) are shown. In the center, blue and white segments represent gene presence and absence, respectively. The line graph at the bottom presents the percentage of isolates containing specific genes, starting from core-genome genes, and showing a rapid decrease with accessory genes.
