# Supplementary material for: Whole-genome-based characterization of Campylobacter jejuni from human patients with gastroenteritis collected over an 18 year period reveals increasing prevalence of antimicrobial resistance
Source: Microb Genom. 2023 Feb 21;9(2):mgen000941. doi: 10.1099/mgen.0.000941 (PMC9997746; doi:10.1099/mgen.0.000941)
Supplement: Supplementary material 3 [file mgen-9-941-s003.pdf]

A

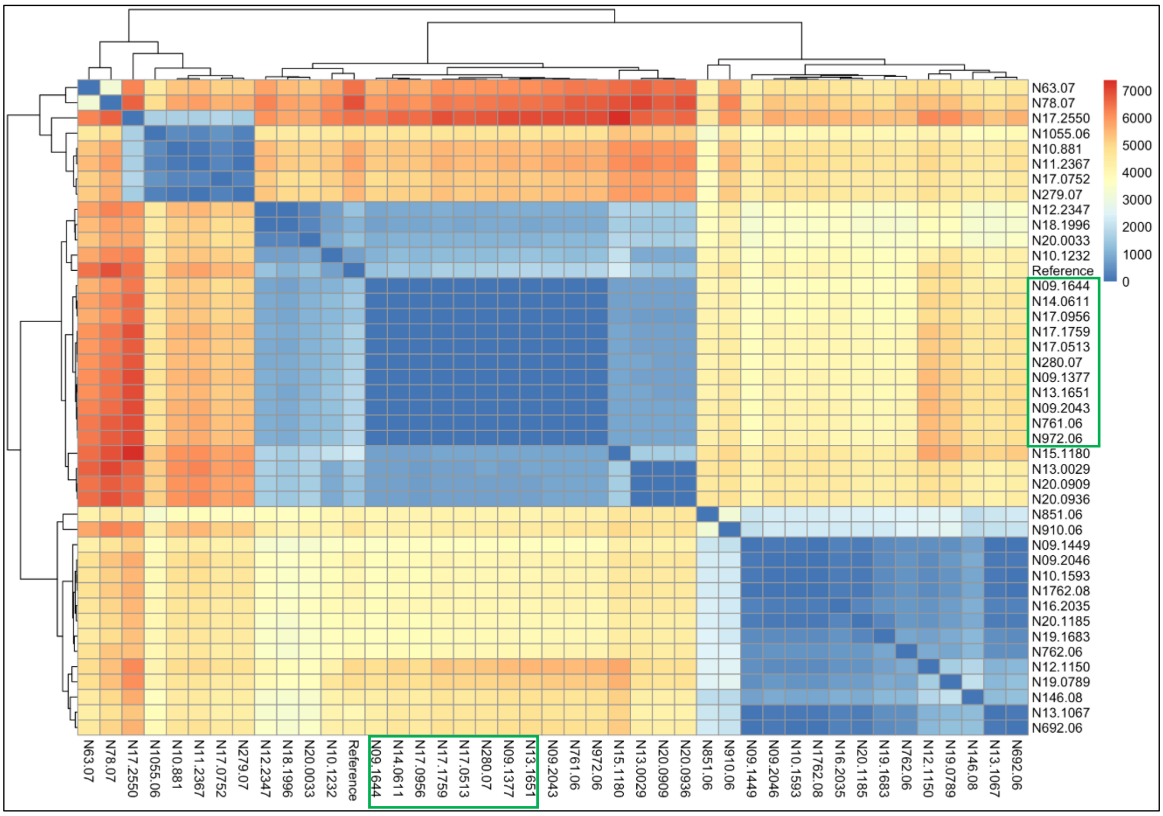
7000
6000
5000
4000
3000
2000
1000
0
N63.07
N78.07
N17.2550
N1055.06
N10.881
N11.2367
N17.0752
N279.07
N12.2347
N18.1996
N20.0033
N10.1232
Reference
N09.1644
N14.0611
N17.0956
N17.1759
N17.0513
N280.07
N09.1377
N13.1651
N09.2043
N761.06
N972.06
N15.1180
N13.0029
N20.0909
N20.0936
N851.06
N910.06
N09.1449
N09.2046
N10.1593
N1762.08
N16.2035
N20.1185
N19.1683
N762.06
N12.1150
N19.0789
N146.08
N13.1067
N692.06

B

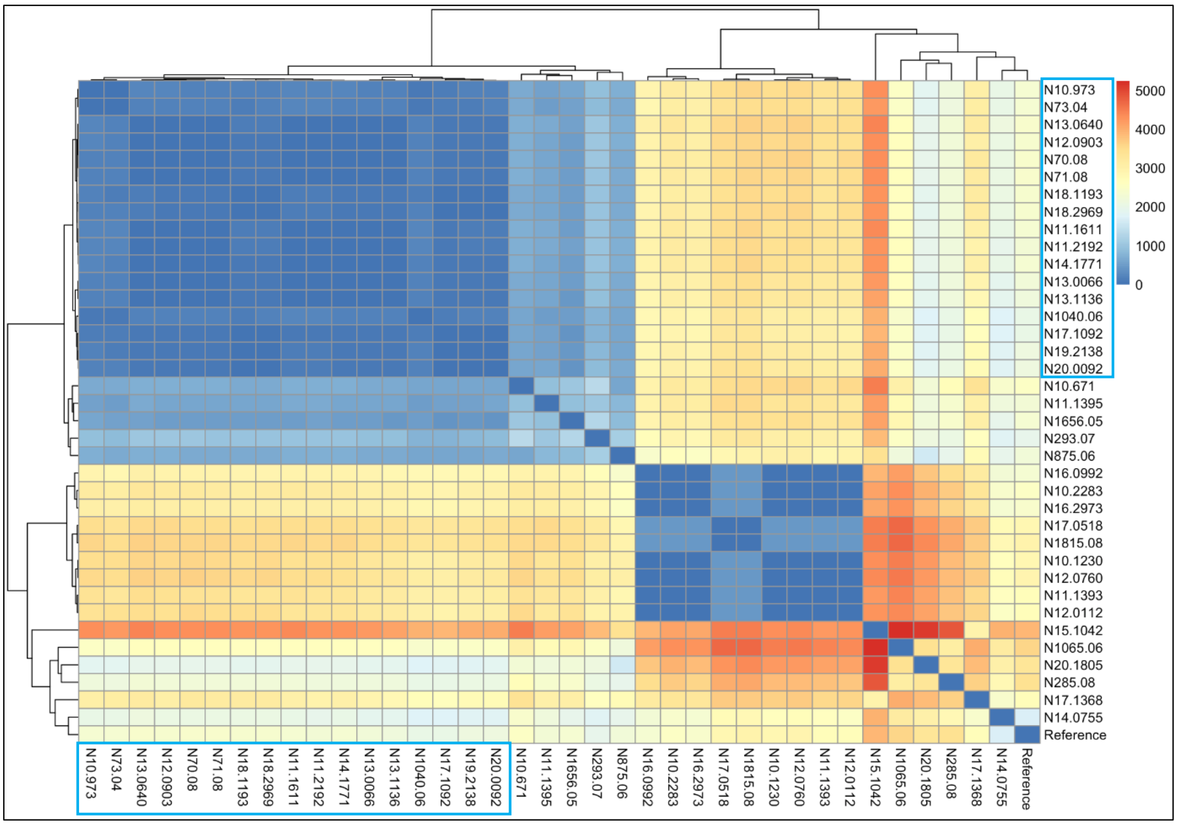
5000
4000
3000
2000
1000
0
N10.973
N73.04
N13.0640
N12.0903
N70.08
N71.08
N18.1193
N18.2969
N11.1611
N11.2192
N14.1771
N13.0066
N13.1136
N1040.06
N17.1092
N19.2138
N20.0092
N10.671
N11.1395
N1656.05
N293.07
N875.06
N16.0992
N10.2283
N16.2973
N17.0518
N1815.08
N10.1230
N12.0760
N11.1393
N12.0112
N15.1042
N1065.06
N20.1805
N285.08
N17.1368
N14.0755
Reference

Supplementary Figure 3. The trimmed reads of all genomes from two of the dominant groups, namely ST-21 (**A**) and ST-50 (**B**), were mapped using Snippy to related complete reference genomes obtained from public repositories. Strain CAMSA2002 (NCBI accession no. GCF_017352015.1) and strain NCTC12658 (NCBI accession no.GCF_019754215.1) were chosen for ST-21 and ST-50 respectively. Pairwise SNP distance matrixes from core genome alignments were computed using snp-dists (version 0.8.2; https://github.com/tseemann/snp-dists) and were used to draw heatmaps combined with the corresponding dendrograms using the pheatmap package (https://github.com/raivokolde/pheatmap). Strains categorized a Cluster A within the ST-21 group and Cluster B in ST-51 are highlighted in green and light-blue respectively.
